# Exogenously-Sourced Ethylene Positively Modulates Photosynthesis, Carbohydrate Metabolism, and Antioxidant Defense to Enhance Heat Tolerance in Rice

**DOI:** 10.3390/ijms23031031

**Published:** 2022-01-18

**Authors:** Harsha Gautam, Mehar Fatma, Zebus Sehar, Noushina Iqbal, Mohammed Albaqami, Nafees A. Khan

**Affiliations:** 1 Plant Physiology and Biochemistry Laboratory, Department of Botany, Aligarh Muslim University, Aligarh 202002, India; harshagautam99@gmail.com (H.G.); meharfatma30@gmail.com (M.F.); seharzebus5779@gmail.com (Z.S.); 2 Department of Botany, Jamia Hamdard, New Delhi 110062, India; naushina.iqbal@gmail.com; 3 Department of Biology, Faculty of Applied Science, Umm Al-Qura University, Makkah 21955, Saudi Arabia

**Keywords:** antioxidant, ethephon, carbohydrate, high-temperature stress, photosynthesis

## Abstract

The effect of exogenously-applied ethylene sourced from ethephon (2-chloroethyl phosphonic acid)was studied on photosynthesis, carbohydrate metabolism, and high-temperature stress tolerance in Taipei-309 and Rasi cultivars of rice (*Oryza sativa* L.). Heat stress increased the content of H_2_O_2_ and thiobarbituric acid reactive substances (TBARS)more in Rasi than Taipei-309. Further, a significant decline in sucrose, starch, and carbohydrate metabolism enzyme activity and photosynthesis was also observed in response to heat stress. The application of ethephon reduced H_2_O_2_ and TBARS content by enhancing the enzymatic antioxidant defense system and improved carbohydrate metabolism, photosynthesis, and growth more conspicuously in Taipei-309 under heat stress. The ethephon application enhanced photosynthesis by up-regulating the *psbA* and *psbB* genes of photosystem II in heat-stressed plants. Interestingly, foliar application of ethephoneffectively down-regulated high-temperature-stress-induced elevated ethylene biosynthesis gene expression. Overall, ethephon application optimized ethylene levels under high-temperature stress to regulate the antioxidant enzymatic system and carbohydrate metabolism, reducing the adverse effects on photosynthesis. These findings suggest that ethylene regulates photosynthesis via carbohydrate metabolism and the antioxidant system, thereby influencing high-temperature stress tolerance in rice.

## 1. Introduction

Climate change has emerged as one of the world’s most pressing environmental issues. On account of climate change, extreme weather events, such as increasing temperatures, changing rainfall patterns, and a rise in the frequency of droughts and floods, are expected to become more common [1]. In turn, significant effects on agriculture and food security are likewise expected [2]. According to the Intergovernmental Panel on Climate Change (IPCC), the average global temperature may rise by 1.8–4°C by the end of the twenty-first century and another 1.1–6.4°C in the following century [3]. High-temperature stress is one of the most severe environmental stresses for crops, as it limits plant growth, development, and productivity in a number of ways [4,5]. High-temperature stress during a crop’s reproductive period has a presumably negative impact on its productivity [6]. High-temperature stress reduces leaf chlorophyll content and degrades chlorophyll molecules due to the generation of reactive oxygen species (ROS) [7,8]. It is also associated with thylakoid membrane damage and destacking, decreased photosystem II (PS II) activity, and the dissociation of oxygen-evolving complexes [9,10]. The other impacts of high temperatures include stomatal closure, reduced carbon dioxide flow, hampered metabolic activities, reduced activity of ribulose 1,5-bisphosphate carboxylase/oxygenase (Rubisco),and photosynthesis [11,12]. High-temperature stress has been observed to evoke a decrease in sucrose content and increases in reducing sugars and leaf soluble sugar content [13]. Furthermore, high temperatures negatively impact enzymes involved in starch and chlorophyll biosynthesis [14,15].

Rice (*Oryza sativa* L.), also known as Asian rice, is an edible starchy cereal grain that belongs to the *Poaceae* family [16]. It is the most-consumed staple food for over half of the world’s population, especially in Asia and Africa; as such, it is a valuable food crop that provides food security to many countries worldwide [17,18]. With the world’s population being expected to reach 10 billion by 2050, the demand for rice will likely rise faster than the demand for other crops [19]. Currently, rice is grown at temperatures higher than the ideal (28/22 °C) in most major areas. As such, any further increase in mean temperature, or episodes of high temperature during sensitive stages of plant development, may dramatically reduce rice yields [19]. In fact, each degree Celsius of rise in the global mean temperature will reduce global rice yield by 3.2% in the absence of CO_2_ fertilization, efficient adaptation, and genetic improvement [20]. Moreover, by 2030, 16% of rice-growing areas are expected to be exposed to temperatures above the critical threshold for at least five days during the reproductive period [21]. Thus, high-temperature stress is wellestablished as posing a significant threat to rice production, stability, and yield [22].

Of the various possible cellular and metabolic responses that can occur in plants, the impairment of carbon metabolism and utilization appears to be the major cause of abnormal development and yield loss under high temperatures [23]. In particular, high-temperature stress has been identified to reduce photosynthesis, resulting in low sucrose in leaves and decreased phloem export [24]. Importantly, non-structural carbohydrates (NSCs) are the major source of carbon and energy in plants, and they also play key signaling roles in several physiological processes [25]. Thus, under high-temperature stress, decreased leaf photosynthesis reduces carbohydrate supply, even as the increase in dark respiration and photorespiration causes increased carbohydrate demand, potentially disrupting the plant’s carbon balance [26].

Phytohormones are naturally occurring organic compounds and endogenous signal molecules involved in nearly every aspect of plant development, growth, and defense processes [27,28]. A number of previous studies have shown that phytohormones play important roles in plant responses to high-temperature stress [8,29,30,31].In particular, the exogenous application of phytohormones has significantly reduced heat-induced damage and increased plant heat tolerance [32,33]. The exogenous application of phytohormones could boost plant efficiency under high-temperature stress by protecting main physiological processes by reducing photoinhibition, lowering lipid peroxidation, accumulating osmolytes, stimulating antioxidant mechanisms, and activating the transcription of various genes encoding heat shock proteins [34].

Ethylene (C_2_H_4_) is a simple, gaseous phytohormone that plays a crucial role in controlling plant growth and development. It is produced endogenously by plants and can be supplied exogenously; however, ethylene gas may not be conveniently available for laboratory use. Several chemicals can be used as stand-ins for ethylene [35], of which a widely-used example is the organophosphorus compound ethephon (2-chloroethylphosphonic acid, Cl-CH_2_-CH_2_-PO_3_H_2_). It is applied to plants in the form of a mist or sprayand penetrates through the stomata and cuticles to the apoplastswhere, at pH 5.0 and above, it decomposes to form ethylene, chloride, and phosphate [36,37]. The ethylene so released affects the treated plant’s physiological processes, enhancing growth and photosynthesis, enzymatic and non-enzymatic antioxidant production, and stomatal opening under different abiotic stresses [38,39,40,41]. In *Brassica juncea* exposed to salt, ethylene combined with sulfur increased photosynthetic nitrogen-use efficiency, as well as proline and antioxidant metabolism [42,43]. Ethylene is known to enhance chlorophyll content in non-senescing leaves while causing degradation in mature leaves [44]. It is involved in defense by suppressing ROS formation via the activation of flavonol production in guard cells, and consequently reduces abscisic acid (ABA)-induced stomatal closure [45]. In addition, ethylene regulates carbohydrate partitioning [44], and cross-talk between glucose and ethylene has been explored [46]. In *Hevea brasiliensis*, ethylene increased latex production, enhanced the transcript abundance of sucrose transporters [47], and altered *TREHALOSE-6-PHOSPHATE SYNTHASE* expression [48]. Although individual studies have reported the effects of ethylene on sucrose, trehalose, or glucose metabolism, to date we have found no study that explores its role in regulating the overall carbohydrate metabolism (content and enzymes) under heat stress. Here, we investigated the effects of ethephon on the enzymatic antioxidant system, biochemical indicators, expression of ethylene biosynthesis genesas well as *psbA* and *psbB* genes, which encode core PSII proteins, proline content, and photosynthesis in rice cultivars exposed to high-temperature stress. We observed that the application of 1.6 mM ethephon significantly increased growth and yield characteristics, enhanced activity of the antioxidant system, modulated carbohydrate metabolism, and increased the protection of photosynthesis.

## 2. Results

### 2.1. Selection of Ethephon Concentration to Maximize Photosynthesis and Growth

The influence of ethephon on net photosynthesis (*Pn*), stomatal conductance (*Gs*), and intercellular CO_2_concentration (*Ci*) varied with the concentration in both cultivars. After the application of 1.6 mM ethephon, *Pn*, *Gs*, and *Ci* increased by 50.5%, 51.5%, and 29.8% in Taipei-309 and 43.4%, 48.1%, and 16.4% in Rasi, respectively, relative to control plants; hence, Taipei-309 showed greater increases overall. Except for the 2.0 mM treatment, ethephon increased Soil Plant Analysis Development (SPAD) values in both cultivars.The best effect was observed with 1.6 mM, which increased SPAD values by 17.8% in Taipei-309 and 14.1% in Rasi. Moreover, of the concentrations studied, 1.6 mM ethephon enhanced the Fv/Fm ratio maximally relative to the control plants (Table 1).

**Figure 1:**
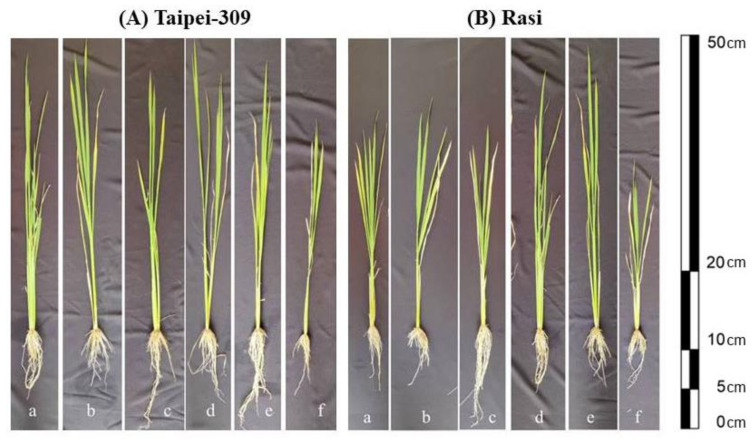
The influence of foliage treated with ethephon (a) 0 mM, (b) 0.4 mM, (c) 0.8 mM, (d) 1.2 mM, (e) 1.6 mM, and (f) 2.0 mM at 15 DAS on plant morphology in rice (*Oryza sativa* L.) cultivars (**A**) Taipei-309 and (**B**) Rasi at 30 DAS. DAS, days after sowing.

Ethephon treatment also altered the morphology of the tested rice cultivars. Except for the 2.0 mM dosage, foliar application of ethephon boosted all growth parameters; the most significant increase was observed when plants were sprayed with 1.6 mM ethephon. In particular, relative to control plants, the application of 1.6 mM ethephon increased plant height, shoot fresh weight, root fresh weight, shoot dry weight, and root dry weight by 14.1%, 7.97%, 12.3%, 15.9%, and 16.1% in Taipei-309 and 10.0%, 6.07%, 10.5%, 13.5%, and 12.2%in Rasi, respectively, compared to the control. In contrast, treatment with 2.0 mM ethephon reduced the said parameters in both cultivars, with a greater reduction in Rasi (Table 2, Figure 1).

### 2.2. Ethephon Reduces the Oxidative Stress Associated with High-Temperature Stress

The extent of cellular damage caused by high-temperature-induced oxidative stress was determined in terms of hydrogen peroxide (H_2_O_2_) content, and membrane damage in terms of thiobarbituric acid reactive substances (TBARS) content. Relative to control plants, high-temperature stress significantly enhanced H_2_O_2_ and TBARS content by 197.4% and 100.0% in Taipei-309 and by 221.3% and 140.4% in Rasi, respectively; that is, cultivar Rasi showed more substantial increases in both H_2_O_2_ and TBARS content. Exogenously applied ethephon at 1.6 mM maximally mitigated the temperature-induced oxidative stress, significantly reducing H_2_O_2_ and TBARS content in both rice cultivars, although moreconspicuously in Taipei-309 (Table 3).

### 2.3. Ethephon Accelerates Antioxidant Enzyme Activity under High-Temperature Stress

Antioxidant enzyme activities increased significantly in response to high temperatures with or without the application of exogenous ethephon. Relative to control plants, the high-temperature treatment stimulated the antioxidant enzymes superoxide dismutase (SOD), ascorbate peroxidase (APX), and glutathione reductase (GR) by 29.2%, 43.7%, and 30.8% in Taipei-309 and 21.8%, 37.2%,and 22.0% in Rasi, respectively. Meanwhile, treatment with ethephon increased the activities of SOD, APX, and GR in Taipei-309 by 62.2%, 111.1%, and 81.1% and in Rasi by 56.0%, 99.1%, and 77.9%, respectively. Finally, when exposed to the combination of high temperatures and ethephon treatment, a significant enhancement of these ROS-scavenging enzymes was observed, with increases of 101.5%, 175.5%, and 125.1% in Taipei-309 and 97.9%, 166.1%, and 113.9% in Rasi, respectively (Table 3).

### 2.4. Effect of Ethephon on Proline, Nitrogen, and Sulfur Content, as Well as Nitrate Reductase Activity under High-Temperature Stress

High-temperature stress triggered proline accumulation, with proline content increased by 33.0% in Taipei-309 and 30.5% in Rasi compared to control plants. Exogenously-supplied ethephon led to even more substantial increases in proline content, about 80.1% in Taipei-309 and 75.4% in Rasi. Plants treated with exogenous ethephon and high-temperature stress accumulated more proline, with an observed increase of 111.0% in Taipei-309 and 100.8% in Rasi (Table 4).

The impacts of high-temperature stress and ethephon treatment on nitrogen (N) and sulfur (S) content, as well as nitrate reductase (NR) activity, in rice leaves are presented in Table 4. Compared to control plants, high-temperature stress reduced N and S content and NR activity alike, by 25.1%, 21.8%, and 22.1% in Taipei-309 and by 27.3%, 32.1%, and 25.2%, in Rasi, respectively. Liekwise, plants treated with ethephon also showed a significant increase in N and S content as well as NR activity, with increases of 34.3%, 16.1%, and 38.7% in Taipei-309 and 31.5%, 13.0%, and 33.9% in Rasi, respectively. Similarly, treatment with high-temperature stress combined with ethephon increased N and S content and NR activity by 18.9%, 17.9%, and 22.3% in Taipei-309 and 8.41%, 5.39%, and 19.3% in Rasi, respectively (Table 4).

### 2.5. Ethephon Promotes Photosynthesis, Growth, and Yield Attributes under High-Temperature Stress

**Figure 2:**
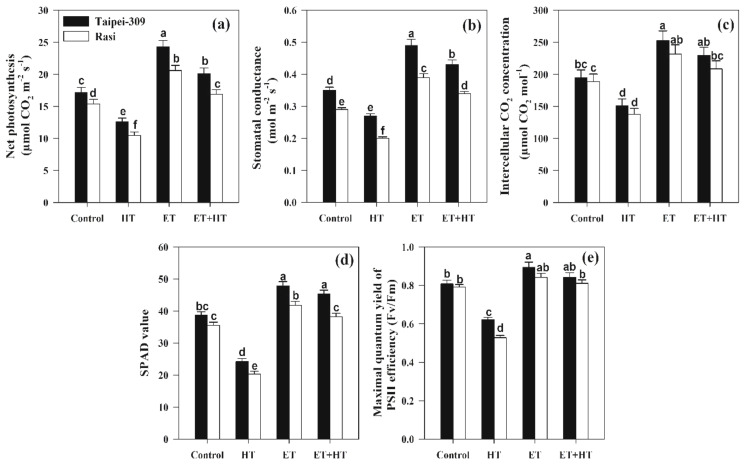
(**a**) Net photosynthesis, (**b**) stomatal conductance, (**c**) intercellular CO_2_ concentration, (**d**) SPAD value, and (**e**) maximal quantum yield of PSII efficiency (Fv/Fm) in Taipei-309 and Rasi cultivars of rice (*Oryza sativa* L.) at 30 DAS. Plants were grown with/without high-temperature (HT) stress and treated with a foliar spray of 1.6 mM ethephon (ET) at 15 DAS. Data are presented as treatment means ± SE (*n* = 4). Bars with the same letters did not differ significantly by the LSD test at *p* < 0.05. DAS, days after sowing.

High-temperature stress significantly affected leaf gas-exchange attributes and SPAD values in both rice cultivars (Figure 2a–d). In particular, high-temperature stress decreased *Pn*, *Gs*, *Ci*,and SPAD values relative to control plants by 26.7%, 22.8%, 22.2%, and 37.4% in Taipei-309 and by 31.8%, 31.0%, 27.0%, and 42.8% in Rasi, respectively. Meanwhile, ethephon application resulted in higher values of *Pn*, *Gs*, *Ci*, and SPAD, with increases of 41.2%, 40.0%, 29.8%, and 23.7% in Taipei-309 and 33.7%, 34.4%, 22.9%, and 17.7% in Rasi, respectively. Furthermore, plants subjected to both ethephon and high-temperature treatment demonstrated increases in *Pn*, *Gs*, *Ci*, and SPAD values of 16.8%, 22.8%, 18.0%, and 17.0% for Taipei-309 and 9.74%, 17.2%, 10.6%, and 7.60% for Rasi, respectively. Finally, high-temperature stress significantly reduced PSII activity (Fv/Fm) in both rice cultivars; the application of ethephon inhibited this decrease and improved PSII activity relative to control plants (Figure 2e).

**Figure 3:**
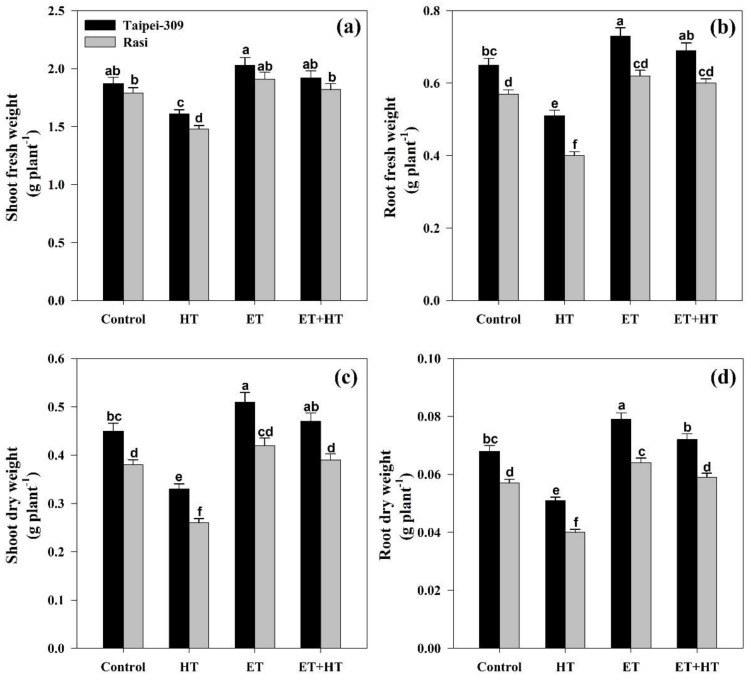
(**a**) Shoot fresh weight, (**b**) root fresh weight, (**c**) shoot dry weight, and (**d**) root dry weight in Taipei-309 and Rasi cultivars of rice (*Oryza sativa* L.) at 30 DAS. Plants were grown with/without high-temperature (HT) stress and treated with a foliar spray of 1.6 mM ethephon (ET) at 15 DAS. Data are presented as treatment means ± SE (*n* = 4). Bars with the same letters did not differ significantly by the LSD test at *p* < 0.05. DAS, days after sowing.

Notably, exposure to high temperatures more severely hampered the growth of the cultivar Rasi than that of Taipei-309; specifically, shoot and root fresh weights and dry weights relative to control plants were lessened by 17.3%, 29.8%, 31.5%, and 29.8% in Rasi, but only by 13.9%, 21.5%, 26.6%, and 25.0% in Taipei-309, respectively. However, plant biomass was enhanced by ethephon application under both normal and high-temperature conditions. With ethephon alone, the observed maximum shoot and root fresh and dry weights increased by 6.70%, 8.77%, 10.5%, and 12.2% in Rasi, and by 8.55%, 12.3%, 13.3%, and 16.1% in Taipei-309, respectively. Meanwhile, the application of ethephon in conjunction with high temperatures improved the same measuremetns by 1.67%, 5.26%, 2.63%, and 3.50% in Rasi, and by 2.67%, 6.15%, 4.44%, and 5.88% in Taipei-309, respectively (Figure 3a–d).

As with the growth and physiochemical parameters, high-temperature stress also reduced the yield characteristics of the tested rice cultivars. In particular, the number of tillers per plant, the number of panicles per plant, panicle length, the number of grains per panicle, and 1000-grain weight were reduced by 18.9%, 20.7%, 9.33%, 18.0%, and 14.9% in Taipei-309, and by 20.6%, 23.1%, 10.1%, 19.3%, and 19.5% in Rasi, respectively, compared to control plants. In contrast, ethephon treatment at 1.6 mMin the absence of the stress condition increased said parameters by 25.2%, 24.6%, 15.1%, 19.8%, and 23.1% in Taipei-309, and by 21.8%, 21.7%, 12.5%, 17.8%, and 21.1% in Rasi, respectively, compared to the control. Subsequently, ethephon application in the context of high-temperature stress was effective in decreasing the deleterious effects of the stress. The tillers per plant, panicles per plant, panicle length, grains per panicle, and 1000-grain weight were increased by 16.8%, 15.5%, 10.6%, 15.3%, and 15.4% in Taipei-309 and by 13.7%, 13.0%, 9.66%, 12.8%, and 13.2% in Rasi, respectively, compared to the control (Table 5).

### 2.6. Effect of Ethephon on Total Non-Structural Carbohydrate, Soluble Sugar, and Sucrose Contents When under High-Temperature Stress

**Figure 4:**
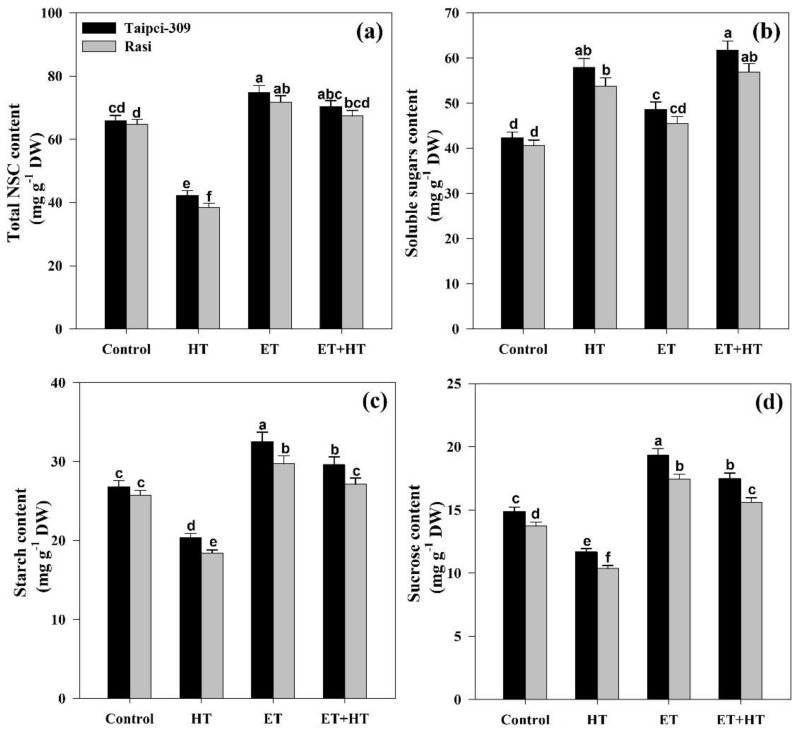
(**a**) Total non-structural carbohydrate (NSC) content, (**b**) soluble sugar content, (**c**) starch content, and (**d**) sucrose content in Taipei-309 and Rasi cultivars of rice (*Oryza sativa* L.) at 30 DAS. Plants were grown with/without high-temperature (HT) stress and treated with a foliar spray of 1.6 mM ethephon (ET) at 15 DAS. Data are presented as treatment means ± SE (*n* = 4). Bars with the same letters did not differ significantly by the LSD test at *p* < 0.05. DAS, days after sowing; DW, dry weight.

This study investigated the effects of high-temperature stress and ethephon treatment on total non-structural carbohydrate (NSC), soluble sugar, and sucrose content in the shoots of selected rice cultivars. Relative to control plants, high-temperature stress significantly reduced total NSC content by 35.8% in Taipei-309 and 40.4% in Rasi. Conversely, ethephon treatment significantly increased total NSC content by 13.5% in Taipei-309 and 10.9% in Rasi under non-stress conditions, and by 6.67% in Taipei-309 and 4.17% in Rasi under high-temperature stress compared to the control (Figure 4a). Meanwhile, high-temperature stress increasedthe soluble sugar content by 36.8% in Taipei-309 and 32.5% in Rasi relative to control. Ethephon treatment also increased soluble sugar content by 14.8% in Taipei-309 and 12.0% in Rasi compared to the control. The combination of high-temperature stress and ethephon treatment further increased soluble sugar content by 45.8% in Taipei-309 and 40.1% in Rasi over the control. Thus, considering soluble sugar content, ethephon treatment enhanced plant sensitivity to high-temperature stress (Figure 4b).

Concerning starch accumulation, high-temperature stress reducedits accumulation by 23.8% in Taipei-309 and 28.4% in Rasi relative to the control. Meanwhile, treatment with ethephon increased starch accumulationby 21.2% in Taipei-309 and 15.5% in Rasi when used alone, and by 10.4% in Taipei-309 and 5.44% in Rasi with combined high-temperature stress compared to the control (Figure 4c). Sucrose content also decreased relative to the controls in plants exposed to high temperatures, by 21.6% in Taipei-309 and by 24.3% in Rasi. Exogenous ethephon significantly increased sucrose content in both circumstances tested: by 29.8% in Taipei-309 and 26.8% in Rasi in the non-stress condition, and by 14.8% in Taipei-309 and 12.0% in Rasi for plants exposed to high temperatures (Figure 4d).

### 2.7. Effect of Ethephon on the Calvin Cycle Enzymes under High-Temperature Stress

**Figure 5:**
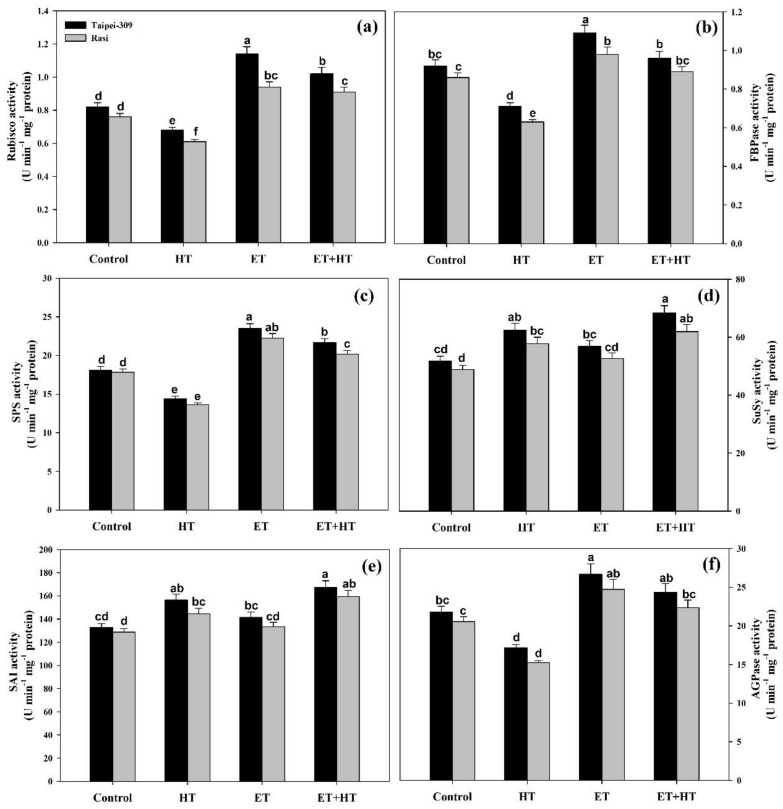
Ribulose 1,5-bisphosphate carboxylase/oxygenase (Rubisco) activity (**a**), fructose bisphosphatase (FBPase) activity (**b**), sucrose phosphate synthase (SPS) activity (**c**), sucrose synthase (SuSy) activity (**d**), soluble acid invertase (SAI) activity (**e**), and ADP-glucose pyrophosphorylase (AGPase) activity (**f**) in Taipei-309 and Rasi cultivars of rice (*Oryza sativa* L.) at 30 DAS. Plants were grown with/without high-temperature (HT) stress and treated with a foliar spray of 1.6 mM ethephon (ET) at 15 DAS.Data are presented as treatment means ± SE (*n* = 4). Bars with the same letters did not differ significantly by the LSD test at *p* < 0.05. DAS, days after sowing.

Exposing plants to high temperatures resulted in decreased activities of ribulose 1,5-bisphosphate carboxylase/oxygenase (Rubisco) and fructose bisphosphatase (FBPase), with reductions of 17.0% and 22.8% in Taipei-309 and 19.7% and 26.7% in Rasi, respectively, relative to the control plants. Meanwhile, treatment with ethephon alone produced significant increases in Rubisco and FBPase activities: by 39.0% and 18.4% in Taipei-309, and by 23.6% and 13.9% in Rasi, respectively. The combination of ethephon and high temperatures likewise resulted in increased Rubisco and FBPase activity by 24.3% and 4.34% in Taipei-309, and by 19.7% and 3.48% in Rasi, respectively, compared to the control (Figure 5a,b).

### 2.8. Effect of Ethephon on Sucrose Metabolism Enzymes under High-Temperature Stress

High-temperature stress had a considerable impact on the activity of sucrose phosphate synthase (SPS), sucrose synthase (SuSy), and soluble acid invertase (SAI), all of which are involved in sucrose synthesis and cleavage. Namely, relative to the controls, SPS activity decreased by 20.6% in Taipei-309 and 23.6% in Rasi, while the activities of SuSy and SAI both increased: SuSy by 20.7% and 18.1%, and SAI by 17.7% and 12.2%, in Taipei-309 and Rasi, respectively. Meanwhile, ethephon treatment enhanced SPS activity in non-stress conditions by 29.6% and 24.8% in Taipei-309 and Rasi, respectively, and also in heat-stressed plants by 19.5% and 13.1% in Taipei-309 and Rasi, respectively, compared to the control. The exogenous application of ethephon also enhanced the activities of SuSy and SAI under non-stress conditions: SuSy by 9.93% and 7.79% and SAI by 6.53% and 3.52% in Taipei-309 and Rasi, respectively, compared to the control. Appropriately, maximum SuSy and SAI activities were achieved with ethephon treatment of plants under high-temperature stress.SuSy activity increased by 32.1% and 26.7%, and SAI activity by 26.0% and 23.5%, in Taipei-309 and Rasi, respectively, over the control (Figure 5c–e).

### 2.9. Effect of Ethephon on Starch Content and ADP-Glucose Pyrophosphorylase Activity

The effect of ethephon on ADP-glucose pyrophosphorylase (AGPase) activity in the rice cultivars was investigated under non-stress and stress conditions. High-temperature stress significantly reduced AGPase enzyme activity relative to the controls by 21.1% in Taipei-309 and 25.8% in Rasi.In contrast, treatment with ethephon alone significantly increased AGPase activity by 22.3% in Taipei-309 and 20.3% in Rasi. Likewise, the combination of high temperatures and ethephon treatment increased AGPase activityby 11.6% in Taipei-309 and 8.86% in Rasiover the control (Figure 5f).

### 2.10. Effect of Ethephon on the Expression of Genes Encoding Core PSII Proteins

**Figure 6:**
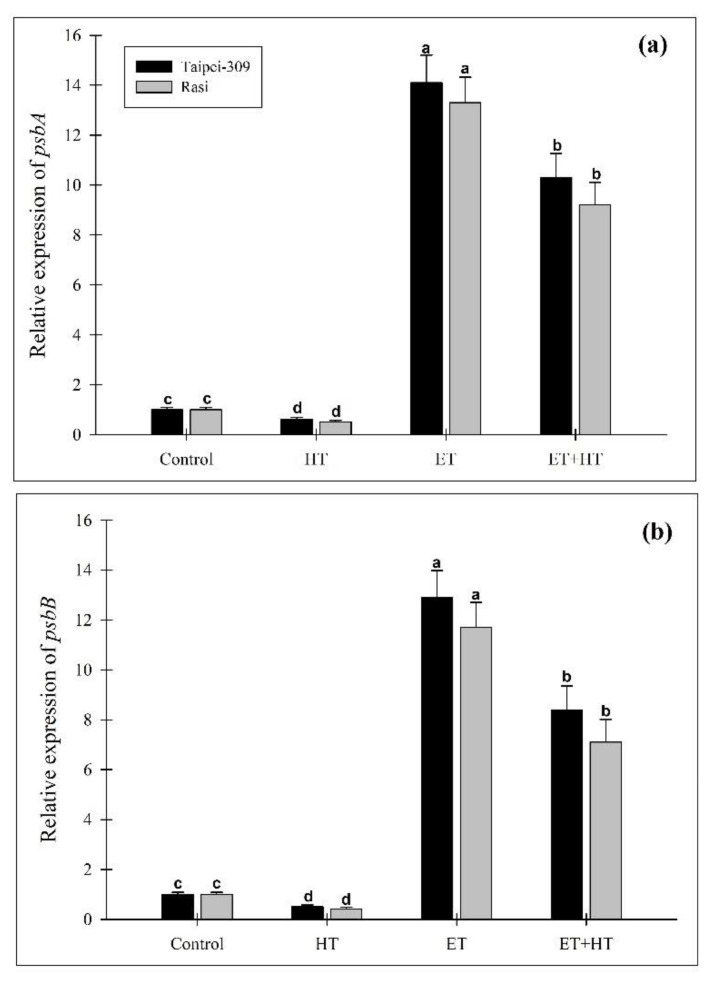
Relative expression of the genes *psbA* (**a**) and *psbB* (**b**) in leaves of Taipei-309 and Rasi cultivars of rice (*Oryza sativa* L.) at 30 DAS. Plants were grown with/without high-temperature (HT) stress and treated with a foliar spray of 1.60 mM ethephon (ET) at 15 DAS. Data are presented as treatment means ± SE (*n* = 4). Bars with the same letters did not differ significantly by the LSD test at *p* < 0.05. DAS, days after sowing.

The exogenous application of ethylene is known to influence the photosynthetic efficiency of PSII proteins and the expression of their encoding genes [46]. Here, the expression of two genes involved in the photosynthetic system, *psbA* and *psbB,* encoding D1 protein and CP47, respectively, were analyzed to further investigate the protective function of ethylene against high-temperature stress. Under normal conditions, we found that ethephon application significantly increased the expression of *psbA* and *psbB* relative to the controls; meanwhile, high-temperature stress reduced their expression. However, in plants exposed to both high temperatures and ethephon, the expression of *psbA* and *psbB* was remarkably increased (Figure 6).

### 2.11. Effect of Ethephon on the Relative Expression of ACS and ACO, as Well as Ethylene Evolution, under High-Temperature Stress

**Figure 7:**
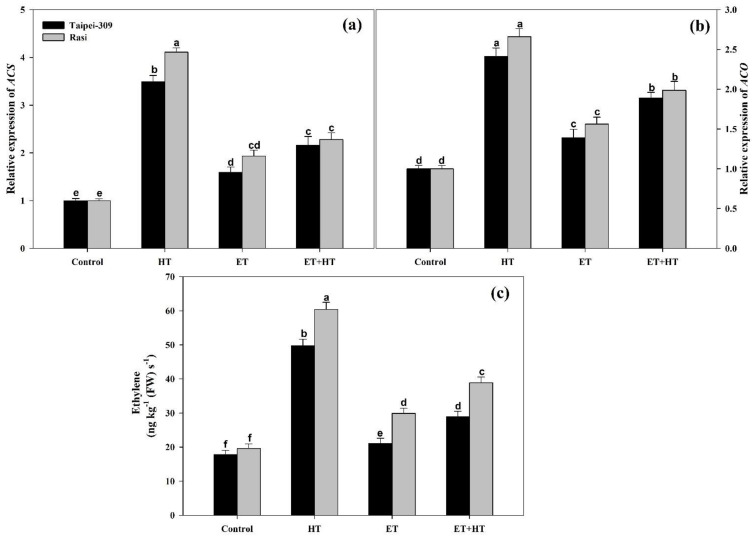
Relative expression of *ACS* (**a**) and *ACO* (**b**), as well as ethylene evolution (**c**),in Taipei-309 and Rasi cultivars of rice (*Oryza sativa* L.) at 30 DAS. Plants were grown with/without high-temperature (HT) stress and treated with a foliar spray of 1.6 mM ethephon (ET) at 15 DAS. Data are presented as treatment means ± SE (*n* = 4). Bars with the same letters did not differ significantly by the LSD test at *p* < 0.05. *ACS*, 1-aminocyclopropane carboxylic acid synthase; *ACO*, 1-aminocyclopropane carboxylic acid oxidase; DAS, days after sowing.

The effect of high-temperature stress and ethephon treatment on the relative expression of the ethylene biosynthesis genes 1-aminocyclopropane-1-carboxylic acid (ACC) synthase (*ACS*) andACCoxidase (*ACO*) in the leaves was prominent. In both cultivars, high-temperature treatment significantly increased the relative expression of *ACS* and *ACO*. However, ethephon treatment of plants subjected to high-temperature stress caused a decrease in the expression of *ACS* and *ACO* compared to high-temperature stress alone (Figure 7a,b).

When applied independently, high-temperature stress and ethephon each increased ethylene biosynthesis in both cultivars, with Taipei-309 having the largest increase in response to both treatments. However, ethephon supplementation to heat-stressed plants decreased ethylene production in both cultivars, with reductions of 68.2% in Taipei-309 and 65.3% in Rasi relative to the high-temperature treatment alone (Figure 7c).

## 3. Discussion

Among the ever-changing elements of the natural environment, one of the most damaging is the steadily increasing ambient temperature. Global climatic conditions are expected to see future temperature increases of 1.5–5.8 °C, leading to global warming that threatens agricultural production [49]. High-temperature stress in plants is defined as an increase in temperature such that a key threshold is surpassed for an extended length of time, causing irreparable damage to plant growth and development processes [50]. This stress disrupts the integrity of membranes, proteins, and cytoskeleton structure, as well as the effectiveness of cellular enzymatic activities, thereby obstructing essential physiological processes and causing metabolic imbalances [51]. One of the main effects of high-temperature stress is the excessive formation of ROS, which leads to oxidative stress in the affected cells [13,49]. High temperatures also alter the gene-level expression of osmoprotectants, detoxifying enzymes, transporters, and regulatory proteins, all of which are engaged in the direct defense against high-temperature stress at the molecular level [52]. In the present study, we examined the impact of different concentrations of ethephon on photosynthetic and growth parameters in the Taipei-309 and Rasi rice cultivars. Foliar treatment of rice plants with 1.6 mM ethephon significantly increased growth characteristics and photosynthesis. The application of 2.0 mM ethephon, on the other hand, negatively impacted growth parameters and photosynthesis in both cultivars (Table 1 and Table 2). Similarly, an earlier study has also shown that mustard plants maximally benefitted with 1.6 mM ethephon treatment, exhibiting the greatest growth, photosynthesis, and Naccumulation in response to this concentration. Further, ethephon concentrations above 1.6 mM inhibited growth, photosynthesis, and N accumulation in mustard plants [53].

Ethylene, a gaseous hormone, is required for plant growth and development, as well astolerance to abiotic stresses such as high temperatures [54]. Indeed, ethylene is a key regulator of abiotic stress responses in plants; namely, responses to abiotic stresses are linked to ethylene accumulation at various concentrations, which, in turn, impacts growth and development [39,55]. Ethylene signaling in plants also contributes to reducing oxidative stress and enhancing thermo-tolerance by preserving chlorophyll content and mitigating heat-stress-induced adversity [56]. Likewise, exogenous ethylene treatment (such as with ethephon) is known to activate various stress-related proteins critical for thermo-tolerance, chiefly by maintaining the functional integrity and stability of plant cells [57]. However, we know relatively little about how ethylene affects carbohydrate metabolism and photosynthesis in the context of high-temperature stress. The current study focused on the effects of ethylene, given as ethephon, in rice cultivars subjected to high-temperature stress. In plant cells, ethephon decomposes through hydrolysis to form ethylene, chloride, and phosphate [36,37]. We found that in rice plants, the ethylene so released regulates carbohydrate accumulation and antioxidative enzyme activity, improving the photosynthetic capabilities of treated plants. Figure 7 depicts ethylene biosynthesisin response to foliar ethephon treatment under heat stress; treatment increased ethylene production in both cultivars tested. Notably, maximum ethylene generation was achieved in heat-stressed plants; this was stress ethylene, and adversely affected plant growth. Several prior studies have described stress-induced ethylene formation and the importance of optimal ethylene levels [43,46]. The observed stress ethylene was reduced by ethephon application, which brought ethylene levels to an optimum and influenced the physiological and metabolic changes associated with high-temperature stress. Plants release stress ethylene under heat-stress conditions by the same mechanism that produces ethylene for normal development. However, the functions of normal and stress ethylene differ considerably. The onset of stress ethylene causes inhibitory effects in plants, but exogenously sourced ethylene at the optimal concentration (1.6 mM) applied to heat-stressed plants initiates protective functions, such as the induction of enzymatic and non-enzymatic antioxidants to scavenge ROS by increasing ethylene sensitivity. This helps reduce the stress and formation of stress ethylene, and plants benefit from the functions of optimal ethylene [58].

In this study, high-temperature stress was observed to enhance oxidative stress in the tested rice cultivars, as indicated by elevated H_2_O_2_ and TBARS levels; the same measurements were lowered in plants treated with ethephon. The activities of the enzymatic antioxidants SOD, APX, and GR also increased in plants exposed to high-temperature stress. However, this response was insufficient to alleviate the temperature-induced oxidative stress. Treatment of heat-stressed plants with ethylene significantly increased antioxidant enzyme activities, with consequent scavenging of H_2_O_2_to minimize oxidative stress (Table 3). Previous reports support that ethylene (sourced from ethephon) increases the activity of antioxidant enzymes such as superoxide dismutase, peroxidases, glutathione/ascorbate reductases, and catalases, which detoxify excess ROS [41,42,59]. Further, antioxidant enzymes like SOD, APX, and GR are essential for detoxifying free radicals and H_2_O_2_ produced during stressful conditions [60]. Accordingly, the elevated H_2_O_2_ and TBARS contents associated with high-temperature stress were greatly reduced by applying ethephon, particularly in the cultivar Taipei-309. This demonstrates the efficacy of ethylene in stress reduction. Ethephon treatment of *Brassica juncea* L. exposed to zinc stress likewise reduced electrolyte leakage and TBARS levels according to Khan and colleagues [61]. Similarly, Wu and Yang [56] found that ethylene treatment of rice seedlings under heat stress alleviated heat-induced oxidative damage by lowering MDA content and electrolyte leakage, and increasing antioxidative enzymes. Ethylene also reduced oxidative stress in mustard plants exposed to cadmium stress by lowering H_2_O_2_ and TBARS levels and increasing photosynthetic capacity [40]. Our results support that, in rice cultivars, enzymatic antioxidant systems (SOD, APX, and GR) are activated by high temperatures to limit oxidative damage, and ethylene treatment further boosts the up-regulationof these antioxidant systems.

Additionally, we found that proline levels increased dramatically in high-temperature-stressed rice, and that ethephon treatment further raised proline content, which could relate to plant mechanisms for coping with high temperatures (Table 4). According to previous studies, the exogenous application of ethephon to nickel-stressed [41] and zinc-stressed [61] plants releases ethylene, which increases proline levels. Proline accumulation may be a sign of stress tolerance [62], as it protects enzymes by stabilizing the structure of proteins like Rubisco and shielding membrane structures [63]. We also observed higher nitrogen allocation to leaves in response to ethephon treatment as a result of increased nitrate reductase activity, which improved photosynthesis in heat-stressed plants (Table 4). Notably, plants deprived of S may experience a significant drop in photosynthetic efficiency [64,65].

Photosynthesis is the most fundamental physiological process in plants, providing vital energy for growth and metabolism. Damage to photosynthetic components can reduce a plant’s overall photosynthetic capacity, whether temporarily or permanently [66]. This study investigated the effect of ethylene on inhibiting high-temperature-induced photosynthetic reduction. We observed that high-temperature stress reduced stomatal conductance, the intercellular CO_2_ concentration, net photosynthesis, SPAD value, and the Fv/Fm ratio (Figure 2). In this context, one cause of decreased photosynthesis is heat-stress-induced stomatal closure, which alters the intercellular CO_2_ concentration [67]. When the tested rice cultivars were given exogenous ethephon, we observed increases in the SPAD value and photosynthetic traits; similar, but lesser, increases were observed with ethephon treatment of plants under high-temperature stress. These findings are consistent with previous studies in which ethylene was used to maintain chlorophyll content and thermo-tolerance in rice seedlings under high-temperature stress [56]. Exogenous ethylene also reportedly improved the photosynthetic efficiency and growth of mustard plants under cadmium stress [40].

High-temperature stress during reproductive development in *Triticum aestivum* causes decreased photosynthesis and leaf area, as well as losses in shoot and grain mass plus sugar content [68]. In the present study, rice growth parameters (namely the fresh and dry weights of shoots and roots) were likewise negatively affected by high-temperature stress, with greater impacts observed in the cultivar Rasi (Table 2). This decline in plant growth could be due to changes in carbohydrate metabolism and nitrogen assimilation [69]. However, all evaluated growth and yield parameters were significantly enhanced by the exogenous ethephon application. This is consistent with a prior report that, relative to untreated seedlings, ethylene-precursor-treated rice seedlings had a higher survival rate and less fresh weight loss when exposed to high temperatures [56]. Cicchino and colleagues [70] demonstrated that providing ethephon (250 g ha^−1^) to *Zea mays* plants under high-temperature stress (35 and 48 °C) optimized phenological events and crop biomass. In the present study, high-temperature stress also negatively affected the yield characteristicsof the tested rice cultivars, more prominently in Rasi, and the application of ethephon, again, significantly reduced these detrimental effects (Table 5). All told, our findings imply that ethylene improves photosynthesis and growth, enabling a spectacular recovery of yield in rice plants subjected to heat stress.

High-temperature stress influences photosynthesis by modulating the activity of enzymes involved in carbon metabolism, sucrose synthesis, and starch accumulation, chiefly via regulating genes in the carbon metabolism pathway [71]. For example, reduced photosynthetic efficiency could be caused by the suppression of Calvin cycle enzymes such as Rubisco [72], the activities of which have been reported to change in response to temperature stress [73,74]. High-temperature stress also reduces the activity of sucrose phosphate synthase, ADP-glucose pyrophosphorylase, and invertases, all of which are involved in sucrose and starch synthesis [74].

We also observed that high-temperature stress dramatically increases soluble sugars in the rice cultivars, whereas levels of sucrose, total non-structural carbohydrates, and starch were lower than the controls (Figure 4). The observed increase in soluble sugar was due to the increased activity of sucrose breakdown enzymes such as sucrose synthase and soluble acid invertase. Sucrose is hydrolyzed into hexoses (glucose and fructose) by invertases, such as soluble acid invertase, and sucrose synthase provides cells with fuel for respiration and also supplies carbon and energy for the synthesis of a variety of compounds [75]. Thus, the observed heat-stress-induced increase in soluble acid invertase and sucrose synthase activities was associated with increased total soluble sugars and decreased sucrose content. Rice plants exposed to high temperatures also exhibited decreased sucrose phosphate synthase activity, consequently contribing to the reduced sucrose level. Thus, high-temperature stress can disrupt sugar (carbohydrate) metabolismin a range of crops, causing stunted plant growth and development. Here, we found that high-temperature stress reduced the activities of Rubisco and FBPase in rice plants, and that supplementation of ethylene as ethephon ameliorated this effect (Figure 5).

High-temperature stress has previously been reported to negatively impact the activity of sucrose phosphate synthase, but enhance the activity of sucrose synthase and invertases [76]. Likewise, *Cucumis sativus* exposed to high-temperature stress exhibited increased soluble acid invertase and neutral invertase activity [77]. High invertase activity in high-temperature conditions boosts sugar metabolism and provides a substrate for glycolysis and the tricarboxylic acid cycle [78]. In heat-sensitive genotypes, a significant change in sucrose phosphate synthase activity during high temperatures has been reported, resulting in decreased sucrose synthase activity [79]. Conversely, according to Lafta and Lorenzen [80], increased expression of sucrose phosphate synthase genes at high temperatures promotes sucrose production, improving plant tolerance to mild heat stress. In the present study, we observed two mechanisms that compound in lowering sucrose content: reduced activity of its synthesizing enzyme (sucrose phosphate synthase) and increased activity of its degrading enzymes (sucrose synthase and soluble acid invertase). Similarly, ethylene treatment was found to increase sucrose content through both enhancing sucrose phosphate synthase activity and reducing sucrose synthase and soluble acid invertaseactivities. In addition, ethylene also reduced oxidative stress by increasing the activity of antioxidative enzymes resulting in increased photosynthesis and sucrose content. Importantly, increased sucrose content reduces osmotic stress because of its potential role as an osmolyte.

The sugars produced by plants during photosynthesis serve as energy metabolism substrates and the precursors of polysaccharides such as starch and cellulose [81]. Moreover, sugars such as glucose, sucrose, fructan, raffinose, and trehalose serve not only as structural components of cells and metabolic resources, but also as signal transduction molecules that regulate many genes involved in plant growth and development, and additionally act as protectants in plant responses to abiotic stimuli such as high temperatures [50]. Plants employ starch and fructan as energy sources and glucose replacements in unfavorable situations [82]; thus, the depletion of carbohydrate reserves and plant starvation has been seen under prolonged high-temperature stress [74]. Of the various plant carbohydrates, sugars like glucose and fructose are more closely linked to plant heat tolerance [83]. Metabolic profile analysis has revealed that plants exposed to a combination of heat and drought stress accumulated sugars (such as glucose, fructose, sucrose, trehalose, and maltose), which helped to preserve cell turgor, stabilize cell membranes, and inhibit protein breakdown [84]. According to Marias and colleagues [85], glucose and fructose were more greatly accumulated in the expanding leaves of *Coffea arabica* under high-temperature stress because these sugars provide the fundamental building blocks for lignin, cellulose, and other structural carbohydrates required to construct the cell walls of those leaves. Similarly, Wahid and Close [86] observed that the accumulation of soluble sugars in *Saccharum officinarum* has a significant impact on heat tolerance. Sucrose in particular, which has been identified as the most abundant carbohydrate in leaves, is able to scavenge hydroxyl radicals in vitro [87]. Here, we observed soluble sugar to increase in rice plants under high-temperature stress, and this accumulation was further increased with ethephon treatment. Thus, ethylene treatment increases sucrose and starch levels, which has the multifaceted consequences of lowering the oxidative stress caused by high temperatures, as well as providing a better environment for photosynthetic enzymes and improved photosynthesis, while also sustaining osmotic resilience and supplying energy for growth and development.

In addition to impacting sugar content, high-temperature stress can impair the activity of starch synthesis enzymes and starch content [88]. In rice plants under drought stress, Prathap and Tyagi [89] reported that the starch content of leaves dropped, and low starch content was positively correlated with low activity of AGPase, one of the key rate-limiting enzymes in starch biosynthesis. Various other studies support that AGPase activity is inhibited by high temperatures in several crop plants, resulting in lower starch biosynthesis [88,90]. Likewise, we found that heat-stressed rice plants exhibited reduced starch accumulation and lowered AGPase activity; however, exogenous ethephon increased both starch levels and AGPase activity in plants under high-temperature stress. Ethephon treatment also boosted sucrose hydrolysis during high-temperature stress by increasing sucrose synthase and soluble acid invertase activity. Other abiotic stresses (such as drought) have also been reported to dramatically lower starch accumulation, probably due to decreased activity of starch-synthesizing enzymes (i.e., AGPase, soluble starch synthase, and starch branching enzyme) [91]. The ameliorative effect of ethylene observed here occurred through increased levels of antioxidative enzymes and regulation of carbohydrate metabolism. The increased Rubisco and sucrose phosphate synthase activity, as well as the reducedsoluble acid invertase and sucrose synthase activity, promoted sucrose and starch content with consequent increases in photosynthesis and growth.

Ethylene regulates the expression of a number of genes, including photosynthetic proteins, redox system enzymes, and senescence-associated genes [92,93]. Through transcriptomic analysis studies, Smet and colleagues [94] determined that the expression of photosynthetic genes was altered in ethylene-treated *Arabidopsis* plants exposed to high CO_2_ concentrations. Salt stress in wheat plants was found to suppress genes encoding PSII proteins; however, pretreatment with ethylene caused those same genes to beup-regulated instead [46]. Here, the effect of ethylene on genes encoding core PSII proteins in rice plants subjected to high-temperature stress was investigated. Our results revealed that in response to ethephon treatment, *psbA* and *psbB* expression increased significantly (Figure 6). This possibly explains the improved stability of PSII under high-temperature stress when treated with ethylene; in particular, greater transcription of *psbA* would support the repair and renewal of the D1 protein it encodes, which is degraded during high-temperature stress. To an extent, ethephon treatment also induced CP47 transcription and improved reaction center function. In higher plants, ethylene biosynthesis is mediated by two major enzymes, ACC synthase and ACC oxidase, which control the amount of ACC produced and the speed of its oxidative degradation, respectively [95]. According to the results of the present study, *ACC synthase* and *ACC oxidase* gene expression were considerably up-regulated in heat-exposed rice plants. However, the expression of *ACC synthase* and *ACC oxidase* was down-regulated in response to ethephon treatment compared to that of high-temperature conditions with the corresponding changes in ethylene (Figure 7).

## 4. Materials and Methods

### 4.1. Reagents

Reagents and chemicals were purchased from Sigma-Aldrich (St. Louis, MO, USA) and S.D. Fine Chem. Limited (Mumbai, India).

### 4.2. Plant Material, Growth Conditions, and Experimental Design

Two cultivars of rice (*Oryza sativa* L.), Taipei-309 and Rasi, were obtained from the Indian Agricultural Research Institute, New Delhi. These cultivars were selected for study on account of Taipei-309 being tolerant and Rasi non-tolerant of elevated temperatures, as determined after screening for changes in photosynthesis, growth, and yield parameters relative to controls according to our previous study [8]. The healthy seeds of each cultivar were separately surface sterilized with HgCl_2_ (0.01%) for 2 min, and then washed thoroughly with distilled water. The sterilized seeds were soaked in distilled water for 12–24 h and then incubated at 30 °C. After incubation, the seeds were sown in 25-cm diameter earthen pots filled with acid-washed sand (4 kg). The pots were sterilized before sowing, and all pots were placed in an environmental growth chamber (Khera-Instruments, New Delhi) with a day/night regime of 16/8h, photosynthetically active photon flux density (PPFD) of 200 µmol m^−2^ s^−1^ at plant level, temperature of 28 °C in the light and 22 °C in the dark, and relative humidity of 65 ± 5%. Ten seeds of each cultivar were sown per pot; after thinning, three seedlings were kept in each pot. The plants raised in sand culture were supplemented with Hoagland nutrient solutionsas reported earlier [8].

A concentration of 0, 0.4, 0.8, 1.2, 1.6, or 2.0 mM ethephon (2-chloroethyl phosphonic acid), an ethylene-releasing compound, was applied on the foliage of plants at 15 days after sowing (DAS), and the resultant effects on photosynthetic and growth parameters investigated. The molarity of the ethephon stock solution was determined using the following constants: specific gravity 1.2, purity 40%,and molecular mass 144.5. The different concentrations of ethephon were prepared by diluting the stock solution with double distilled water. As 1.6 mM ethephon increased photosynthesis and growth the most, that dosage was chosen for further study. In another experiment, plants were subjected to a high-temperature stress treatment ten days after sowing, in which pots were placed in a growth chamber at 40 °C for 6 h per day for 15 days, with a day/night cycle of 16/8 h and relative humidity of 65 ± 5%. The plants were then allowed to recover at normal temperature (28 °C) and remained so for the rest of the experimental period (five days; total time period was 30 days). Control plants were kept at 28 °C for the duration of the experiment (30 days). At 15DAS, a hand sprayer was used to apply ethephon (30 mL at 1.6 mM) to the foliage of both high-temperature-treated and non-treated plants. Corresponding control groups were sprayed with deionized water. A surfactant teepol (0.5%) was added with the control and ethephon treatment solutions. Ethephon releases ethylene and phosphate upon hydrolysis. The yield of phosphate is equivalent to ethylene so that one mol of phosphate arises from one mol of ethephon [96,97]. Therefore, the phosphate available from 2.0 mM ethephon was adjusted in the other treatments as single super phosphate to nullify the effects of P. Treatments were arranged in a completely randomized block design considering two factors, and each treatment was replicated four times (*n* = 4). Plants were sampled at 30 DAS to record the various parameters of interest.

### 4.3. Measurement of Photosynthetic Characteristics

Gas exchange measurements were performed on a fully-expanded third leaf for each treatment. Measurements for net photosynthesis, stomatal conductance, and intercellular CO_2_ concentration were taken on an infrared gas analyzer (CID-340, Photosynthesis System, Bio-science, Camas, WA, USA). All measurements were taken between 1100 and 1200 h at an atmospheric CO_2_ concentration of 380 ± 5 µmol mol^−1^, relative humidity of 70%, photosynthetically active radiation of 780 µmol m^−2^ s^−1^_,_ and air temperature of 28 °C. The SPAD value was determined non-destructively with a SPAD chlorophyll meter (502 DL PLUS, Spectrum Technologies, Plainfield, IL, USA). Leaf attributes were measured on the mid-portion of the leaf blade, and those measurements were made in the early morning hours.

The maximal quantum yield of PSII efficiency, as given by Fv/Fm, was determined with a chlorophyll fluorometer (Junior-PAM, Heinz Walz, GmbH, Effeltrich, Germany) from the uppermost fully expanded leaves. The maximum fluorescence (Fm) and variable fluorescence (Fv), the latter given as (Fm–Fo), were assessed after dark-adaptation for 30 min using leaf clips. Initial fluorescence (Fo) was determined by the exposure of dark-adapted leaves to a weak modulated measuring beam having a PPFD of 0.1 µmol photons m^−2^ s^−1^, while Fm was obtained from a saturating pulse (>6000 µmol photons m^−2^ s^−1^). The maximal quantum yield of PSII efficiency was estimated by the equation Fv/Fm = (Fm–Fo)/Fm.

### 4.4. Determination of Oxidative Stress Indicators

ROS accumulation was assessed by measuring H_2_O_2_ content following the method described by Okuda et al. [98]. To estimate H_2_O_2_, 500 mg of fresh leaf tissue was homogenized in ice-cold 200 mM perchloric acid (HClO_4_), followed by centrifugation at 1200 × *g* for 10 min. Next, the supernatant was neutralized with 4 M KOH. The homogenate was further centrifuged at 500 × *g* for 3 min for the removal of insoluble potassium perchlorate. For the determination of H_2_O_2_, the reaction mixture (1.5 mL) contained 1 mL of the eluate, 80 µL of 3-methyl-2-benzothiazoline hydrazone, 400 µL of 12.5 mM 3-(dimethylamino) benzoic acid in 0.375 M phosphate buffer (pH 6.5), and 20 µL of peroxidase (0.25 unit). The reaction was initiated with the addition of peroxidase at 25 °C, and the resulting increase in absorbance was estimated at 590 nm on a spectrophotometer.

Lipid peroxidation was determined by measuring the content of thiobarbituric acid reactive substances (TBARS) as described by Dhindsa et al. [99]. Fresh leaf samples (500 mg) were ground in 0.25% 2-thiobarbituric acid (TBA) in 10% trichloroacetic acid (TCA) using a mortar and pestle. The mixture was heated at 95 °C for 30 min, rapidly cooled in an ice bath, and centrifuged at 10,000 × *g* for 10 min. To 1 mL of the resulting supernatant, 4.0 mL of 20% TCA containing 5% TBA was added. The absorbance of the supernatant was read at 532 nm and corrected for non-specific turbidity by subtracting the absorbance of the same at 600 nm. TBARS content was calculated using an extinction coefficient of 155 mM^−1^ cm^−1^.

### 4.5. Estimation of Proline Content

Proline content in leaves was estimated using the method described by Bates et al. [100]. The details of the method are given in Supplementary File S1.

### 4.6. Determination of Nitrogen and Sulfur Content

Nitrogen content in leaves was measured through the Kjeldahl digestion method as described by Lindner [101]; S content in leaves was determined using the turbidimetric method of Chesnin and Yien [102]. The details of these methods are given in Supplementary File S1.

### 4.7. Determination of Nitrate Reductase Activity

Leaf nitrate reductase activity was assayed following the method of Kuo et al. [103]. The details of the method are given in Supplementary file S1.

### 4.8. Determination of Soluble Sugars, Sucrose, Starch and Total Non-Structural Carbohydrate Content

Soluble sugars and sucrose content were measured using the method of Xu et al. [104]. Starch estimation was performed using the method of Kuai et al. [105]. The details of the methods are given in Supplementary File S1.

### 4.9. Determination of Growth and Yield Parameters

Rice plants were uprooted and washed with water to remove adhering sand, then blotted with a soft paper towel to remove excess moisture. The above ground and underground parts of plants were separated using scissors, and the roots were carefully cleaned with deionized water. Fresh weights of shoots and roots were determined immediately after the washing process using an electronic balance. The dry weights of shoots and roots were recorded after drying the samples in a hot air oven (80 °C) for 72 h, such that a constant weight was achieved. The number of tillers per plant was counted manually at weekly intervals and at harvest. Panicle appearance was documented daily. The number of panicles per plant, panicle length, the number of grains per panicle, and 1000-grain weight were all determined manually at harvest time.

### 4.10. Determination of Antioxidant Enzyme Activities

Fresh leaf tissue (200 mg) was collected from third leaves and quickly ground in ice-cold extraction buffer containing potassium-phosphate buffer (100 mM, pH 7.0), 0.05% (*v*/*v*) Triton X-100, and 1% (*w*/*v*) polyvinylpyrrolidone (PVP). The homogenates were then centrifuged at 15,000 × *g* for 20 min at 4 °C. The clear supernatant obtained after centrifugation was used for assaying enzymatic activities. Assays utilized APX extraction buffer supplemented with 2 mM ascorbate.

SOD activity in protein extracts was determined according to Beyer and Fridovich [106] and Giannopolitis and Ries [107], which relies on the inhibition of the photochemical reduction of nitro blue tetrazolium (NBT). Briefly, 5.0 mL of reaction mixture containing 5 mM HEPES (pH 7.6), 0.1 mM EDTA, 50 mM Na_2_CO_3_ (pH 10.0), 13 mM methionine, 0.025% (*v*/*v*) Triton X-100, 63 µmol NBT, and 1.3 µmol riboflavin was mixed with the enzyme-containing extract. The reactants were then placed in bright light (360 µmol m^−2^ s^−1^) for 15 min, while a corresponding control was not illuminated to allow for the correction of background absorbance. One unit of SOD is defined as the amount of enzyme needed to inhibit NBT reduction by 50% (as measured by the absorbance at 560 nm).

APX activity was measured according to the method of Nakano and Asada [108]. The assay mixture (1.0 mL) contained phosphate buffer (50 mM, pH 7.0), 0.1 mM EDTA, 0.5 mM ascorbate, 0.1 mM H_2_O_2_, and enzyme extract, and was observed at 290 nm for 1 min using a spectrophotometer. A decrease in absorbance was observed as soon as the reaction was started (i.e., upon the addition of H_2_O_2_). An extinction coefficient of 2.8 mM^−1^ cm^−1^ was used when computing APX activity. One unit of APX is defined as the amount necessary to decompose one µmol of substrate per min at 25 °C.

GR activity was estimated following the method of Foyer and Halliwell [109], in which the glutathione (GSH)-dependent oxidation of nicotinamide adenine dinucleotide phosphate (NADPH) is monitored at 340 nm. The reaction mixture (3.0 mL) contained phosphate buffer (25 mM, pH 7.8), 0.5 mM GSSG, 0.2 mM NADPH, and the enzyme extract. The reaction was initiated upon the addition of GSSG, and a decreasing trend in absorbance was immediately evident. An extinction coefficient of 6.2 mM^−1^ cm^−1^ was used when quantifying GR activity. One unit of enzyme is defined as the amount necessary to decompose one µmol of NADPH per min at 25 °C.

### 4.11. Determination of Carbohydrate Metabolic Enzyme Activities

The activity of Rubisco was determined spectrophotometrically according to the method of Usuda [110], specifically monitoring NADH oxidation at 30 °C via absorbance at 340 nm during the conversion of 3-phosphoglycerate to glycerol 3-phosphate after the addition of the enzyme extract to the assay medium. Protein was estimated according to Bradford [111] using bovine serum albumin as the standard. The details of the methods are given in Supplementary File S1.

The activity of fructose-1,6-bisphosphatase (FBPase) was measured spectrophotometrically by monitoring absorbance at 340 nm according to the method reported by Rao and Terry [112]. The details of the procedure are provided in Supplementary File S1.

The activity of the enzymes sucrose phosphate synthase, sucrose synthase, and soluble acid invertase was evaluated using the Kalwade and Devarumath [113] method. The activity of ADP-glucose pyrophosphorylase was determined using the method of Kleczkowski et al. [114], which relies upon the absorbance at 340 nm during the conversion of NADP to NADPH. The details of these methods are given in Supplementary File S1.

### 4.12. RNA Isolation and cDNA Synthesis

Total RNA was isolated from rice leaves using TRIzol reagent (Ambion, Life Technologies, Austin, TX, USA) according to the manufacturer’s instructions. The extracted RNA was quantified using a Nanodrop spectrophotometer (Thermo Scientific, Waltham, MA, USA). To ensure the integrity of the RNA, each sample was run on an agarose formaldehyde gel [115]. For both the control and treated samples, first-strand cDNA was made from 1 µg of total RNA and a reaction mixture containing 20 U/µL Moloney murine leukemia virus reverse transcriptase (MuMLV) (Fermentas, Waltham, MA, USA), incubated at 42 °C for 50 min and at 70 °C for 10 min. The reverse transcription reaction was carried out using 2.5 µM Oligo (dT) 18 primer (Fermentas, USA) and 10 mM dNTPs. Primers for gene expression analysis were designed using online primer designing software (IDT), and the cDNA sequences of selected genes were obtained from NCBI.

### 4.13. Quantitative Real-Time PCR Analysis

Real-time PCR (RT-PCR) was performed in 96-well reaction plates (Roche, Mannheim, Germany) containing a 20-µL reaction mixture of 10X reaction buffer, 2 mM dNTPs, 1 mM MgCl_2_, 0.35 µM each of forward and reverse primers, 1 µL SYBR Green (10X), 10 µg cDNA template, and 5 U Taq polymerase on a thermal cycler (Lightcycler 480 II, Roche, Germany). All quantifications were normalized to an actin DNA fragment amplified by β-actin forward and β-actin reverse primers, and the actin gene was used as an internal control for evaluating the per-gene efficiency of RT-PCR. PCR cycling conditions were as follows: denaturation at 95 °C for 3 min, 40 cycles of 95 °C (20 s), 66 °C (1 min) and 72 °C (1 min), and a final extension at 72 °C for 5 min. The amplified product was resolved on a 1.2% agarose gel. Amplicon specificity was verified by melting curve analysis (60 to 95 °C). All reactions were performed as three biological replicates (with three technical replicates of each), using gene-specific primers and actin primers as an internal control. The primer pairs used for quantitative RT-PCR are listed in Supplementary Table S1. The results were presented as the expression of the gene of interest in relation to the internal control in the treated sample compared with corresponding values in the untreated control.

### 4.14. Estimation of Ethylene Evolution

Ethylene evolution in leaves was assessed using a gas chromatograph according the process described previously by Fatma et al. [43].

### 4.15. Statistical Analysis

Obtained data were examined statistically using a two-way analysis of variance (ANOVA) with SPSS software version 17.0 for Windows and presented as treatment means ± SE (*n* = 4). The least significant difference (LSD) was calculated for data found to be significant at *p* < 0.05. Bars with the same letter are not significantly different by the LSD test at *p* < 0.05.

## 5. Conclusions

This study revealed that ethephon application at a 1.6 mM concentration more effectively ameliorated the high-temperature-stress-inhibited photosynthesis, growth, and yield more conspicuously in the Taipei-309 rice cultivar than the Rasi cultivar. Specifically, ethylene altered the morphological, physiological, and biochemical parameters in heat-stressed Taipei-309 more positively and significantly than Rasi. Exogenous ethephon protected photosynthetic machinery against heat-stress-induced oxidative damage by increasing the activity of enzymatic antioxidants and altering the expression of genes encoding core PSII proteins. It improved growth and yield by modulating carbohydrate metabolism under unfavourable conditions more prominently in Taipei-309. Thus, the results of this study suggested that ethylene could be used to enhance photosynthesis, growth, and yield in the context of heat stress.

## Figures and Tables

**Table 1:** The influence of foliage treated with 0, 0.4, 0.8, 1.2, 1.6, or 2.0 mM ethephon at 15 DAS on net photosynthesis, stomatal conductance, intercellular CO_2_ concentration, SPAD value, and maximal quantum yield of PSII efficiency (Fv/Fm) in Taipei-309 and Rasi cultivars of rice (*Oryza sativa* L.) at 30 DAS. Data are presented as treatment means ± SE (*n* = 4). Data followed by the same letter are not significantly different by the LSD test at *p* < 0.05. DAS, days after sowing.

Ethephon (mM)	Cultivars	Net Photosynthesis (µmol CO_2_ m^−2^ s^−1^)	Stomatal Conductance (mol m^−2^ s^−1^)	Intercellular CO_2_ Concentration (µmol CO_2_ mol^−1^)	SPAD Value	Maximal Quantum Yield of PSII Efficiency (Fv/Fm)
0	Taipei-309	16.8 ± 0.8 ^de^	0.33 ± 0.011 ^efg^	193.8 ± 11.5 ^bcd^	37.5 ± 1.24 ^cde^	0.804 ± 0.020 ^bcd^
	Rasi	15.2 ± 0.7 ^ef^	0.27 ± 0.007 ^hi^	187.2 ± 10.0 ^cd^	34.7 ± 1.15 ^de^	0.791 ± 0.019 ^cde^
0.4	Taipei-309	19.9 ± 0.9 ^c^	0.37 ± 0.015 ^cde^	215.6 ± 14.2 ^abcd^	39.1 ± 1.28 ^cd^	0.830 ± 0.033 ^abc^
	Rasi	18.7 ± 0.8 ^cd^	0.31 ± 0.011 ^fgh^	198.5 ± 11.9 ^bcd^	35.9 ± 1.20 ^de^	0.811 ± 0.032 ^bcd^
0.8	Taipei-309	21.2 ± 1.0 ^bc^	0.42 ± 0.019 ^b^	229.3 ± 15.7 ^ab^	40.9 ± 1.38 ^abc^	0.859 ± 0.021 ^abc^
	Rasi	19.1 ± 0.9 ^cd^	0.35 ± 0.012 ^def^	219.7 ± 14.5 ^abc^	37.2 ± 1.22 ^cde^	0.826 ± 0.020 ^abc^
1.2	Taipei-309	23.6 ± 1.1 ^ab^	0.48 ± 0.021 ^a^	240.7 ± 16.4 ^a^	42.7 ± 1.40 ^ab^	0.871 ± 0.017 ^ab^
	Rasi	20.4 ± 1.0 ^bc^	0.39 ± 0.016 ^bcd^	227.5 ±15.5 ^ab^	38.4 ± 1.26 ^cd^	0.841 ± 0.016 ^abc^
1.6	Taipei-309	25.3 ± 1.2 ^a^	0.50 ± 0.023 ^a^	251.6 ± 16.5 ^a^	44.2 ± 1.44 ^a^	0.892 ± 0.022 ^a^
	Rasi	21.8 ± 1.0 ^bc^	0.41 ± 0.017 ^bc^	231.9 ± 16.4 ^ab^	39.6 ± 1.32 ^bc^	0.853 ± 0.021 ^abc^
2.0	Taipei-309	14.4 ± 0.7 ^ef^	0.29 ± 0.009 ^gh^	187.8 ± 10.0 ^cd^	33.8 ± 1.17 ^e^	0.776 ± 0.019 ^de^
	Rasi	12.7 ± 0.6 ^f^	0.23 ±0.005 ^i^	176.4 ± 9.7 ^d^	28.5 ± 1.12 ^f^	0.759 ± 0.019 ^e^

**Table 2:** The influence of foliage treated with 0, 0.4, 0.8, 1.2, 1.6, or 2.0 mM ethephon at 15 DAS on plant height, shoot fresh weight, root fresh weight, shoot dry weight, and root dry weight of Taipei-309 and Rasi cultivars of rice (*Oryza sativa* L.) at 30 DAS. Data are presented as treatment means ± SE (*n* = 4). Data followed by the same letter are not significantly different by the LSD test at *p* < 0.05. DAS, days after sowing.

Ethephon (mM)	Cultivars	Plant Height (cm)	Shoot Fresh Weight (g Plant^−1^)	Root Fresh Weight (g Plant^−1^)	Shoot Dry Weight (g Plant^−1^)	Root Dry Weight (g Plant^−1^)
0	Taipei-309	38.2 ± 1.28 ^cd^	1.88 ± 0.05 ^abc^	0.65 ± 0.016 ^cde^	0.44 ± 0.011 ^cd^	0.068 ± 0.0017 ^cd^
	Rasi	35.7 ± 1.10 ^d^	1.81 ± 0.03 ^cd^	0.57 ± 0.014 ^g^	0.37 ± 0.009 ^f^	0.057 ± 0.0014 ^fg^
0.4	Taipei-309	39.5 ± 1.33 ^cd^	1.90 ± 0.06 ^abc^	0.67 ± 0.026 ^bcd^	0.46 ± 0.018 ^bc^	0.070 ± 0.0028 ^c^
	Rasi	36.8 ± 1.15 ^d^	1.84 ± 0.04 ^cd^	0.59 ± 0.023 ^fg^	0.39 ± 0.015 ^ef^	0.059 ± 0.0023 ^ef^
0.8	Taipei-309	41.7 ± 1.36 ^abc^	1.94 ± 0.06 ^abc^	0.68 ± 0.017 ^abc^	0.48 ± 0.012 ^ab^	0.073 ± 0.0018 ^bc^
	Rasi	37.4 ± 1.22 ^d^	1.86 ± 0.04 ^bc^	0.60 ± 0.015 ^efg^	0.40 ± 0.010 ^ef^	0.061 ± 0.0015 ^ef^
1.2	Taipei-309	42.9 ± 1.39 ^ab^	1.99 ± 0.06 ^ab^	0.71 ± 0.014 ^ab^	0.49 ± 0.009 ^ab^	0.077 ± 0.0015 ^ab^
	Rasi	38.7 ± 1.24 ^cd^	1.90 ± 0.06 ^abc^	0.62 ± 0.012 ^defg^	0.41 ± 0.008 ^de^	0.062 ± 0.0012 ^ef^
1.6	Taipei-309	43.6 ± 1.42 ^a^	2.03 ± 0.07 ^a^	0.73 ± 0.018 ^a^	0.51 ± 0.012 ^a^	0.079 ± 0.0019 ^a^
	Rasi	39.3 ± 1.31 ^bcd^	1.92 ± 0.06 ^abc^	0.63 ± 0.015 ^cde^	0.42 ± 0.010 ^de^	0.064 ± 0.0016 ^de^
2.0	Taipei-309	31.4 ± 1.21 ^e^	1.63 ± 0.05 ^de^	0.51 ± 0.012 ^h^	0.33 ± 0.008 ^g^	0.052 ± 0.0013 ^g^
	Rasi	27.5 ± 1.06 ^f^	1.51 ± 0.02 ^e^	0.42 ± 0.010 ^i^	0.26 ± 0.006 ^h^	0.041 ± 0.0010 ^h^

**Table 3:** Content of leaf hydrogen peroxide (H_2_O_2_) and thiobarbituric acid reactive substances (TBARS) and activity of superoxide dismutase (SOD), ascorbate peroxidase (APX), and glutathione reductase (GR) in Taipei-309 and Rasi cultivars of rice (*Oryza sativa* L.)at 30 DAS. Plants were grown with/without high-temperature (HT) stress, and the foliage was treated with 1.6 mM ethephon (ET) at 15 DAS. Data are presented as treatment means ± SE (*n* = 4). The values followed by the same letters did not differ significantly by the LSD test at *p* < 0.05. FW, fresh weight; DAS, days after sowing.

Cultivars	Treatments	H_2_O_2_ Content	TBARS Content	SOD Activity	APX Activity	GR Activity
		(nmol g^−1^ FW)		(U min^−1 ^mg^−1^ Protein)		
Taipei-309	Control	31.8 ± 1.70 ^d^	12.4 ± 0.32 ^de^	7.89 ± 0.32 ^de^	1.35 ± 0.04 ^fg^	0.191 ± 0.005 ^ef^
	HT	94.6 ± 4.30 ^b^	24.8 ± 1.17 ^b^	10.2 ± 0.49 ^c^	1.94 ± 0.09 ^e^	0.25 ± 0.005 ^d^
	ET	22.3 ± 1.32 ^e^	8.2 ± 0.23 ^f^	12.8 ± 0.52 ^b^	2.85 ± 0.12 ^c^	0.346 ± 0.011 ^b^
	ET+HT	44.7 ± 2.10 ^c^	14.1 ± 0.44 ^cd^	15.9 ± 0.56 ^a^	3.72 ± 0.13 ^a^	0.43 ± 0.01 ^a^
Rasi	Control	35.1 ± 1.99 ^d^	13.1 ± 0.37 ^de^	6.92 ± 0.27 ^e^	1.18 ± 0.03 ^g^	0.172 ± 0.004 ^f^
	HT	112.8 ± 5.02 ^a^	31.5 ± 1.22 ^a^	8.43 ± 0.34 ^d^	1.62 ± 0.07 ^f^	0.21 ± 0.005 ^e^
	ET	29.9 ± 1.59 ^de^	11.2 ± 0.28 ^e^	10.8 ± 0.41 ^c^	2.35 ± 0.10 ^d^	0.306 ± 0.009 ^c^
	ET+HT	52.4 ± 3.01 ^c^	15.8 ± 0.60 ^c^	13.7 ± 0.51 ^b^	3.14 ± 0.11 ^b^	0.368 ± 0.010 ^b^

**Table 4:** Content of leaf nitrogen, sulfur, and proline, as well as nitrate reductase activity, in Taipei-309 and Rasi cultivars of rice (*Oryza sativa* L.) at 30 DAS. Plants were grown with/without high-temperature (HT) stress and treated with a foliar spray of 1.6 mM ethephon (ET) at 15 DAS. Data are presented as treatment means ± SE (*n* = 4). The values followed by the same letters did not differ significantly by the LSD test at *p* < 0.05. FW, fresh weight; DW, dry weight; DAS, days after sowing.

Cultivars	Treatments	Nitrogen Content	Sulfur Content	Proline Content (mg g^−1^ FW)	Nitrate Reductase Activity (U min^−1^ mg^−1^ Protein)
		(mg g^−1^ DW)			
Taipei-309	Control	33.8 ± 1.36 ^cd^	4.16 ± 0.22 ^bc^	13.6 ± 0.59 ^ef^	38.0 ± 1.45 ^c^
	HT	25.3 ± 1.28 ^e^	3.25 ± 0.13 ^d^	18.1 ± 0.75 ^d^	29.9 ± 1.30 ^d^
	ET	45.4 ± 1.45 ^a^	4.83 ± 0.28 ^a^	24.5 ± 0.92 ^b^	52.7 ± 1.75 ^a^
	ET+HT	40.2 ± 1.40 ^b^	4.51 ± 0.24 ^ab^	28.7 ± 1.14 ^a^	46.5 ± 1.69 ^b^
Rasi	Control	30.7 ± 1.34 ^d^	3.52 ± 0.17 ^cd^	11.8 ± 0.42 ^f^	33.0 ± 1.34 ^d^
	HT	22.3 ± 1.24 ^e^	2.39 ± 0.10 ^e^	15.4 ± 0.66 ^e^	25.0 ± 1.25 ^e^
	ET	40.4 ± 1.42 ^b^	3.98 ± 0.20 ^bc^	20.7 ± 0.82 ^c^	44.2 ± 1.60 ^b^
	ET+HT	36.2 ± 1.37 ^bc^	3.71 ± 0.19 ^cd^	23.7 ± 0.89 ^b^	39.4 ± 1.49 ^c^

**Table 5:** Number of tillers per plant, number of panicles per plant, panicle length, number of grains per panicle, and 1000 grain weight in Taipei-309 and Rasi cultivars in rice (*Oryza sativa* L.) at harvest. Plants were grown with/without high-temperature (HT) stress and treated with a foliar spray of 1.6 mM ethephon (ET) at 15 DAS. Data are presented as treatment means ± SE (*n* = 4). The values followed by the same letters did not differ significantly by theLSD test at p < 0.05. DAS, days after sowing.

Cultivars	Treatments	No. of Tillers per Plant	No. of Panicle per Plant	Panicle Length (cm)	No. of Grains per Panicle	1000 Grain Weight (g)
Taipei-309	Control	9.5 ± 0.45 ^bc^	7.7 ± 0.36 ^bc^	22.5 ± 0.91 ^ab^	117.6 ± 3.90 ^abc^	19.4 ± 0.97 ^bcd^
	HT	7.7 ± 0.40 ^de^	6.1 ± 0.30 ^de^	20.4 ± 0.85 ^bc^	96.4 ± 2.34 ^de^	16.5 ± 0.65 ^de^
	ET	11.9 ± 0.61 ^a^	9.6 ± 0.47 ^a^	25.9 ± 1.08 ^a^	140.9 ± 4.67 ^a^	23.9 ± 1.33 ^a^
	ET+HT	11.1 ± 0.58 ^ab^	8.9 ± 0.41 ^ab^	24.9 ± 1.04 ^ab^	135.7 ± 4.31 ^ab^	22.4 ± 1.20 ^ab^
Rasi	Control	8.7 ± 0.40 ^cd^	6.9 ± 0.25 ^cd^	20.7 ± 0.75 ^bc^	110.4 ± 2.91 ^bcd^	18.9 ± 0.86 ^cd^
	HT	6.9 ± 0.35 ^e^	5.3 ± 0.20 ^e^	18.6 ± 0.65 ^c^	89.0 ± 1.94 ^e^	15.2 ± 0.51 ^e^
	ET	10.6 ± 0.50 ^ab^	8.4 ± 0.40 ^b^	23.3 ± 0.90 ^abc^	130.1 ± 3.92 ^ab^	22.9 ± 1.23 ^a^
	ET+HT	9.9 ± 0.47 ^bc^	7.8 ± 0.32 ^bc^	22.7 ± 0.87 ^abc^	124.6 ± 3.56 ^bc^	21.4 ± 1.15 ^abc^

## Data Availability

Data are contained within the article and Supplementary Materials.

## Supplementary Materials

The following are available online at https://www.mdpi.com/article/10.3390/ijms23031031/s1.
