# Supplementary material for: Exogenously-Sourced Ethylene Positively Modulates Photosynthesis, Carbohydrate Metabolism, and Antioxidant Defense to Enhance Heat Tolerance in Rice
Source: Int J Mol Sci. 2022 Jan 18;23(3):1031. doi: 10.3390/ijms23031031 (PMC8835467; doi:10.3390/ijms23031031)
Supplement: Supplementary file 1 [file ijms-23-01031-s001.zip › ijms-1506026-supplementary.pdf]

International Journal of
Molecular Sciences

MDPI

Supplementary

Exogenously-Sourced Ethylene Positively Modulates Photosynthesis, Carbohydrate Metabolism, and Antioxidant Defense to Enhance Heat Tolerance in Rice

Harsha Gautam [1], Mehar Fatma[1], Zebus Sehar[1], Noushina Iqbal[2], Mohammed Albaqami[3*] and Nafees A. Khan[1*]

[1] Plant Physiology and Biochemistry Laboratory, Department of Botany, Aligarh Muslim University, Aligarh 202002, India; harshagautam99@gmail.com (H.G.); meharfatma30@gmail.com (M.F.); seharzebus5779@gmail.com (Z.S.)

[2] Department of Botany, Faculty of Chemical and Life Sciences, Jamia Hamdard, New Delhi 110062 India; naushina.iqbal@gmail.com (N.I.)

[3] Department of Biology, Faculty of Applied Sciences, Umm Al-Qura University, Makkah 21955, Saudi Arabia

*Correspondence: naf9.amu@gmail.com; mmbaqami@uqu.edu.sa

Supplementary File S1

Measurement of Nitrate Reductase Activity

Fresh leaf tissue (1.0 g) was ground in liquid nitrogen using chilled mortar and pestle, and then stored at -80°C. Leaf powder was thawed for 10 min at 4°C and was homogenized in a blender in 250 mM Tris-HCl buffer (pH 8.5), containing 10 mM cysteine, 1 mM EDTA, 20 M FAD, 1 mM DTT, and 10% (v/v) glycerol. The homogenate was centrifuged at 10,000 × g for 30 min at 4°C. The assay mixture contained 10 mM KNO_3, 0.065 M HEPES (pH 7.0), 0.5 mM NADH in 0.04 mM phosphate buffer (pH 7.2), and enzyme in a final volume of 1.5 mL. The reaction was initiated by adding NADH. After 15 min the reaction was terminated by adding 1.0 mL of 1 N HCl solution containing 1% sulfanilamide followed by the addition of 1.0 mL of 0.02% aqueous N-(1-napthyl)-ethylenediaminedihydrochloride (NEDD). The absorbance at 540 nm was measured with a spectrophotometer after 10 min. One Unit (U) of the enzyme activity was defined as nmol product formed.

Determination of Nitrogen and Sulfur Content

For determination of leaf nitrogen content, a 10 mL aliquot of the digested material was taken in a 50 mL volumetric flask. To this, 2.0 mL of 2.5 N NaOH and 1.0 mL of 10% Na_2SiO_3 solutions were added to neutralize the excess of acid and to prevent turbidity, respectively. The volume was made up to the mark with de-ionized water. In a 10 mL graduated test tube, 5.0 mL aliquot of this solution was taken and 0.5 mL Nessler's reagent was added. The final volume was maintained with de-ionized water. The contents of the tubes were allowed to stand for 5 min for maximum color development. The optical density (OD) value of the solution was read on a spectrophotometer at 525

nm. Leaf nitrogen content was calculated using graph plotted between different concentrations of ammonium sulphate and OD.

For estimation of leaf sulfur content, oven-dried leaf powder (100 mg) was taken in a 75 mL digestion tube. A (4.0 mL) mixture of concentrated HNO_3 and 60% $HClO_4$ (85:1 v/v) and 7.5 mg of selenium dioxide as catalyst were added in the tube. The mixture was digested and the volume of the colorless solution was made up to 75 mL with deionized water. The interference of silica was checked by filtering the contents of the tube. A 5.0 mL aliquot was pipette out from the digested solution for turbidity development in 25 mL volumetric flask. Turbidity was developed by adding 2.5 mL gum acacia (0.25%) solution, 1.0 g $BaCl_2$ sieved through 40-60 mm mesh and the volume was made up to the mark with deionized water. The contents of 25 mL volumetric flask were thoroughly shaken till $BaCl_2$ completely dissolved and turbidity was allowed to develop for 2 min. The values were recorded at 415 nm within 10 min after the turbidity development. A blank was also run simultaneously after each set of determination, and calculation was done accordingly.

Determination of Proline Content

Fresh leaf tissue (300 mg) was homogenized in 3.0 mL of 3% sulphosalicylic acid, and the homogenate was centrifuged at 11500×g for 12 min. The supernatant filtrate was added to a test tube with 2.0 mL acid ninhydrin and 2.0 mL glacial acetic acid and incubated in water bath at 10°C for 1 h. Later, 4.0 mL toluene was added to the reaction mixture and mixed vigorously with stirrer for 20-30 s, and then left to stand for 5-10 min. Absorbance of the reddish pink upper phase was measured on a spectrophotometer at 520 nm using L-proline as a standard.

Determination of Soluble Sugar, Starch, Total Non-structural carbohydrate, and Sucrose Content

Shoot samples were oven-dried at 80°C and ground to a fine powder. Approximately 100 mg of the dried sample was extracted using 10 mL of 80% ethanol and kept in a water bath at 80-85°C for 30 min. The extract was centrifuged and the supernatant was transferred to a 100 mL volumetric flask, the extraction was repeated three times. Alcohol extract was evaporated on a water bath at 80-85°C. All the three supernatants were pooled in the flask following by addition of distilled water to 100 mL. Aliquot of the extract was used for determination of soluble sugars with anthrone reagent and the absorbance of reaction mixture was monitored at 630 nm using a spectrophotometer.

Dried shoot samples were ground and filtered using a 1 mm sieve. The powdered material (0.1 g) was put into a 10 mL centrifuge tube and mixed with 5 mL of 80% ethanol. The mixture was incubated at 80°C for 30 min in a water bath shaker then centrifuged at 4000×g for 5 min. The pellets were extracted using 80% ethanol. Ethanol was removed through evaporation. In a boiling water bath, the starch in the residue was released with 2 mL of distilled water for 15 min and then cooled to room temperature. Then, starch was

hydrolyzed with 9.2 mol L^{-1} $HClO_4$ (2 mL) for 15 min. The samples were then centrifuged at 4000×g for 10 min after being supplemented with distilled water (4 mL). 4.6 mol L^{-1} $HClO_4$ was used to extract the residue once again (2 mL). The supernatants were retained, combined, and mixed with distilled water until they reached a volume of 25 mL. Using an anthrone reagent and glucose as the standard, the starch concentration was determined spectrophotometrically at absorbance 620 nm. Soluble sugars and starch content expressed as mg g^{-1} dry weight (DW). The non-structural carbohydrate content was the sum of the soluble sugar content and starch content (mg g^{-1} DW).

For sucrose content estimation each reaction comprised 50 mM UDP-Glucose, 50 mM extraction buffer, 10 mM $MgCl_2$, and 200 L of extract in a total volume of 550 L. The reaction was started by incubating the enzyme extract for 30 min at 30°C. The reaction was stopped using 100 L 2 mol L^{-1} of NaOH and heating the solution for 10 min at 100°C to destroy unreacted hexoses and hexose phosphates. After cooling, 1 mL of 0.1% (w/v) resorcin in 95% (v/v) ethanol, and 3.5 mL of 30% (w/v) HCl were added to the solution and incubated for 10 min at 80°C.

Determination of Activity of Rubisco, Fructose 1,6-bisphosphatase, Sucrose Phosphate Synthase, Sucrose Synthase, Soluble Acid Invertase, and ADP-Glucose Pyrophosphorylase

Fresh leaf samples (1.0 g) were used to determine activity of Rubisco. Leaf samples were homogenized in ice-cold extraction buffer containing 0.25 M Tris-HCl (pH 7.8), 0.05 M $MgCl_2$, 0.0025 M EDTA, and 37.5 mg dithiothreitol (DTT) using a pre-chilled mortar and pestle. The homogenate was centrifuged at 10,000×*g* for 10 min at 4°C, and the supernatant was used to measure enzyme activity. The reaction mixture (3.0 mL) contained 100 mM Tris-HCl (pH 8.0), 40 mM $NaHCO_3$, 10 mM $MgCl_2$, 0.2 mM NADH, 4 mM ATP, 5 mM DTT, 1U of glyceraldehyde 3-phosphodehydrogenase, 1U of 3-phosphoglycerate kinase, and 0.2 mM ribulose 1,5-bisphosphate (RuBP). One unit of enzyme is defined as the μmol of substrate converted.

To determine fructose-1,6-bisphosphatase activity, fresh leaf sample (1.0 g) was homogenized in 4 mL of ice-pre-cooled buffer 0.1 mM Hepes-NaOH (pH 7.5), 50 mM $MgCl_2$, 2 mM EDTA, 2% PVP, and 1% β-mercaptoethanol. The homogenates were centrifuged at 15,000×g for 20 min at 4°C, and the supernatant was used to measure enzyme activity. The assay medium contained 30 mM Hepes-KOH (pH 8.2), 5 mM $MgCl_2$, 5 mM DTT, 0.5 mM NADP, 5 mM fructose-1,6-bisphosphate (FBP), and 2 to 4 units per mL each of phosphogluocoisomerase and glucose-6-phosphorus dehydrogenase. The reaction was started by the addition of FBP and the rate was taken 10-15 min after start of assay. One unit (U) of the enzyme activity was defined as μmol of product formed.

At pH 7.5, a sucrose phosphate synthase and sucrose synthase activity assay was carried out at 37°C in the direction of sucrose synthesis. In 200 μL of assay solution, 200 μL of desalted enzyme extract were added. 100 mM Hepes (pH 7.5), 20 mM Glucose-6-phosphate, 4 mM Fructose-6-phosphate, 3 mM UDP-Glucose, 5 mM $MgCl_2$, and 1 mM EDTA were used in the sucrose phosphate synthase assay solution. 50 mM

Hepes (pH 7.5), 15 mM $MgCl_2$, 25 mM Fructose, and 25 mM UDP-Glucose were used in the sucrose synthase activity assay solution. The assay solutions did not contain UDP-Glucose as a control. The reactions were incubated for 60 min for sucrose phosphate synthase and 30 min for sucrose synthase and then immediately stopped by boiling for 3 min. For the spectrophotometric readings at 630 nm, the enzyme reaction volume and freshly prepared reagents were proportionally increased. At 37°C, acid invertase activity was determined by combining 150 μL desalted enzyme extract with an equivalent volume of 1 M sodium acetate (pH 4.5) and 300 μL of 120 mM sucrose solution. One Unit (U) of sucrose phosphate synthase, sucrose synthase, soluble acid invertase was defined as μmol product formed.

Dried leaves (0.5 g) were homogenized in a pre-chilled mortar and pestle at 40C with cold 2 mL of buffer on ice to determine ADP-Glucose pyrophosphorylase. 50 mM 3-N-morpholino propane sulphonic acid (MOPS) pH 7.4, 2 mM $MgCl_2$, 1 mM EDTA, and 2 mM Dithiothritol (DTT) were used in the extraction buffer. The homogenate was then centrifuged at 10,000×g for 10 min at 40°C in a chilled centrifuge. For enzyme analysis, the supernatant was used. The reaction was initiated by adding 200 μL of sodium pyrophosphate (2.5 μmole) to the mixture. The pyrophosphorolytic activity of ADP-Glucose pyrophosphorylase was measured spectrophotometrically at 340 nm by monitoring the rise in absorbance due to NADP to NADPH conversion. One Unit (U) of ADP-Glucose pyrophosphorylase was expressed as nmol of product formed.

Table S1. Primer pairs used for quantitative RT-PCR

S.No.	Gene	Forward Primer	Reverse Primer
1	*psbA*	ATATTGTGGCCGCTCAT	TCCGTTTAGATTGAAAGCCATAG
2	*psbB*	GCCGGAACTATGTGGTATG	GACCAAGCTTCTGATAAACTGAG
3	*ACS*	CTCCTCGAGGCCTACCTCC	GCGTTGTCCCTGAAGCTACC
4	*ACO*	TGTGTACGCCAAGCAGAAGT	AGCAGATTTTGGCGCCTTGA
**Reference Gene Primer Sequences Used for Quantitative RT-PCR**			
1	Actin	GACTGCCAAGACCAGCTCC	CTTCCTAATATCCACGTCGCAC
